# School-Related Stressors and the Intensity of Perceived Stress Experienced by Adolescents in Poland

**DOI:** 10.3390/ijerph182211791

**Published:** 2021-11-10

**Authors:** Maria Kaczmarek, Sylwia Trambacz-Oleszak

**Affiliations:** Institute of Human Biology and Evolution, Faculty of Biology, Adam Mickiewicz University, 61-614 Poznań, Poland; sylwia.trambacz@amu.edu.pl

**Keywords:** adolescents, perceived stress, gender, school, height, weight status, parents, peers

## Abstract

Higher stress reactivity during adolescence is a vulnerability marker of exposure to various environmental stressors. This study aimed to investigate the association between a high level of perceived stress experienced by adolescents and stressful stimuli induced from school environment, peer, and parental relationships. The data used were from a cross-sectional, observational study conducted in a stratified sample of 1846 adolescents (13–18 years) in the Wielkopolska province, Poland. Data were collected through self-administered questionnaires and anthropometric measurements. Perceived stress was assessed using the Perceived Stress Scale (PSS-10). The association of a high level of perceived stress with school-induced exposures was determined using multivariate logistic regression after adjusting for gender, age, height and weight status and interpersonal relationships (STATISTICA 13.1). It was found that girls were over three times more likely than boys to experience a high level of perceived stress. Moreover, girls appeared to be more vulnerable than boys to school-related stressors and weight status, while boys to stressors that can arise from interpersonal relationships. School environment was the only predictor factor of high perceived stress level with a large effect size in both boys (OR = 4.45; 95% CI: 3.11–6.36) and girls (OR = 6.22; 95% CI: 4.18–7.59). Given the findings of the present study, preventive programs are critical to mitigate the effect of stress from school on adolescents’ health and well-being.

## 1. Introduction

Adolescence is the period of transition between childhood and adulthood. It begins with the onset of puberty (the average age is 10 years for girls and 12 for boys) and ends in the mid-20s with the completion of the growth spurt, attainment of adult stature, and achievement of full reproductive maturity [1,2]. The biological, cognitive, psychological, and emotional transformations in body, brain, and behavior during this stage of development interact simultaneously in a transactional fashion to shape pathways from child to adult [3]. Adolescence is also considered a window of heightened vulnerability to intense, acute and/or chronic stress [4]. Consistent with this is a dramatic rise of acute responses to various stressors. Typical sources of stressors in adolescence are physical (abrupt changes in bodily appearance, development of secondary sexual characteristics, sexual maturation), cognitive (perturbations of the maturing brain), social and environmental (school-induced stress, relations with teachers, parents, and peers) life changes that are novel, challenging, and stressful to young people [5,6]. Acute stress responses in young, healthy individuals may be adaptive and typically do not impose a health burden [7]. However, cumulative and chronic stressors may lead to increased emotional distress, antisocial and risky-for-health behaviors, somatic symptoms, can cause elevated blood pressure, weaken the immune system, and generally, can have negative and lasting effects on physical and mental health in adulthood [8,9].

The association between stressors and health burden depends on the nature, number, and persistence of the stressors as well as the individual’s biological vulnerability, psychosocial resources, and learned patterns of coping with stressful situations [10].

One way of understanding better the processes which underpin stressful situations and improving the way to handle stress is through the transactional model of stress and coping proposed by Lazarus and Folkman [11]. This model presents psychological stress as a transaction and accordingly defines it as “a particular relationship between the person and the environment that is appraised by the person as taxing or exceeding his or her resources and endangering his or her well-being” [11] (p. 19). It means that negative effects of stress on individuals’ wellness or functioning depend on the degree they perceive of the threat level of environmental stimuli and their resources being inadequate to cope with these adversities [11]. This subjective feeling or thoughts that an individual has about the general stressfulness of his or her life and the ability to handle such stress underpins his or her perceived stress [12] (p. 1453). Thus, perceived stress is a multidimensional concept that depends on a wide range of causative factors including various personal traits such as individual effectiveness in coping with demands from the environment, baseline psychopathological state, or personality types [13,14].

Findings from epidemiological studies have confirmed the association between perceived stress and environmental stimuli (stressors). Of all environmental stressors, school-induced stress is the most often reported by adolescents [15]. School is for young people a social environment for both learning (stressful educational demands from teachers and high expectations from parents) and interpersonal relationships with teachers and same-aged peers [16]. Distressed students usually report a large amount of homework, stressful assignments and tests, increasing demands and expectations from school, unhealthy competition between classmates, bullying victimization, and permanent tension surrounding academic performance and social relationships [17]. Parents might also put high expectations on their adolescent children and insist that they practice constantly and perform well (the best) in school competitions and other organized activities to satisfy their needs [18]. There is much evidence for believing that unrealistic parents’ expectations create pressure and foster performance anxiety in their adolescent children [19]. Although family is still important in socialization during adolescence, peers play a major role in both social support and companionship. Positive peer relationships are valuable opportunities for building personal and social skills to enhance interpersonal interactions and better adjustment. But negative peer pressure can lead adolescents to engage in risk-taking behaviors or to be lonely. Both are associated with elevated perceived stress [20].

The sudden and rapid physical changes that adolescents go through make bodily appearance an important component of individual self-esteem, psychological health, and willingness to fully take part in school/social activities [21]. Being overweight and obesity were proven to be significantly associated with perceived stress, psychosocial health problems, and the beginning of bullying victimization [22]. On the other hand, the overwhelming prevalence of thin and lean female imagery and strong and lean male imagery portrayed throughout the media in Western societies has created widespread body image concerns among adolescents [23]. This in turn can lead to weight-related body dissatisfaction and unhealthy eating behavior e.g., dieting. There is much evidence on the association between perceived stress and weight status/weight gains with the possible moderating effect of gender (girls being more likely than boys to report higher levels of stress) and the mediating role of weight-related body dissatisfaction [24].

In recent years, the decline of infectious diseases in children and adolescents has placed mental and substance use disorders (MSUDs), often referred to as the new morbidity or millennial morbidity, among the most common causes of their disability [25]. As reported by the World Health Organization, the MSUDs account for 16% of the global burden of disease and injury in 15–19-year-olds although the prevalence estimates from international studies differ widely [26]. Furthermore, depression has been identified as a major contributor to the overall burden of disease and perceived stress has proven to be an important factor affecting depression in adolescents [27]. The latest findings of the Health Behavior in School-aged Children survey (HBSC 2018) revealed that 31% of 15-year-old girls and 15% of 15-year-old boys in European countries reported feeling low more than once a week, and also, 37% of boys and 22% of boys reported feeling nervous more than once a week. In Poland, the corresponding figures were 35% of girls and 17% of boys reporting feeling low and 48% of girls and 41% of boys reporting feeling nervous [28]. The UNICEF report ranked Poland 31st among 41 developed countries on adolescents’ wellbeing and in second place in Europe in terms of the number of suicides and attempted suicide by young people [29]. These worrying figures reflect a dramatic situation of mental health care in Poland, noting that Poland has the fewest child psychiatrists in Europe (only four per 100,000 residents) and spends just 3.7% of its health budget on funding mental health care [29].

Better evaluation of factors that cause most psychological stress to adolescent life is essential for developing interventions for prevention and promotion. However, comprehensive research on this issue is rather scarce, especially with regard to data for Poland. In response to this research gap, the present study was set up with three objectives in mind: (i) to estimate the prevalence of perceived stress among adolescent students, (ii) to determine the set of factors that affect the likelihood of experiencing a high level of perceived stress, and (iii) to evaluate gender differences in perceived stress and associated factors.

## 2. Materials and Methods

### 2.1. Study Design and Participants

Data for this study were derived from the observational, cross-sectional survey conducted in 2015 in the Wielkopolska province, Poland. The study design and study protocol were approved by the Bioethics Commission of the Poznań University of Medical Sciences and the Poznań Board of Education. All examinations were performed in compliance with principles outlined in the Helsinki Declaration [30]. Sample size was calculated using the formula for quantitative variable and a single cross-sectional survey [31]. Sampling procedure was a stratified two-stage cluster sampling method with the probability proportional to size (PPS) sampling technique [32]. For the first sampling stage, lower and upper secondary schools were selected from the list provided by the Ministry of Education for the Wielkopolska province (https://cie.men.gov.pl) (Accessed on 1 March 2011) followed by the selection of classes from the target grade of each participating school. In this procedure, if the number of classes was more than one, the class was randomly selected (e.g., one class out of every three, four and so on). In most villages however, the students were assigned to only one class of each year level group.

Students attending grades 1 to 3 in lower secondary school (LSS) and 1 and 2 in upper secondary school (USS) were enrolled in the survey, but only those whose parents/legal guardians had given a written consent for them to participate. Adolescents aged 16 and above gave consent on their own behalf. Altogether they accounted for 96.7% of the baseline sample. The study criterion for eligibility was chronological age range 13–18 years. Twenty one students had an individual learning path (home schooling) due to acute or chronic conditions and were thereby exposed to different stressors than their peers regularly attending classes. Those students were excluded from the baseline sample.

Examinations were performed by well-trained personnel in school nursery rooms during morning hours and consisted of two parts: self-reported questionnaire in paper-and-pencil version and anthropometric measurements.

### 2.2. Measures

Data were collected through a self-administered questionnaire that consisted of structured questions on background information pertaining to demographic, social, health indicators (BMI body weight status and psychosomatic symptoms), and perceived stress level. Chronological age was calculated in decimal values by subtracting the date of examination from the date of birth. The age groups were divided by years, defined in terms of the whole year. The reported home residence of the participant at the time of the examination was classified according to online national archives available at https://stat.gov.pl (Accessed 4 May 2016) as rural areas (coded 0) which represented villages, i.e., small communities with a population of less than 1000 people mainly engaged in farm work; small town, ≤25,000 people (coded 1); mid-sized city, >25,000 and <100,000 people (coded 2); large city ≥100,000 people (coded 3).

Body height and body weight were measured according to standard procedures [33] with a portable Swiss-made GPM anthropometer to the nearest 1 mm and on a calibrated electronic scale, Precision Health Scale, to the nearest 0.1 kg. Sex and age-adjusted percentile values derived from the national growth references for Polish school-aged children and adolescents were subsequently used to obtain the three stature categories [34]. Height-for-age between the 25th and 75th percentiles was the average height group; less than the 25th percentile was shorter than and greater than the 75th percentile was taller than the average height group. The body weight status was based on the body mass index (BMI) calculated by taking a subject’s weight (kg) and dividing it by his/her height squared (m^2^). Following the IOTF recommendation, Cole’s cut off values were used to determine the weight status [35,36].

### 2.3. Instruments

Perceived stress was measured using the Perceived Stress Scale (PSS), originally designed by Cohen and colleagues [37], translated to Polish and standardized by Juczyński and Ogińska-Bulik [38]. Study participants were asked to assess the extent to which they appraise life events as stressful during the last month. They rated on a 5-point Likert scale: 0 (never), 1 (hardly ever), 2 (sometimes), 3 (quite often) and 4 (very often), the statements such as “In the last month, how often have you been upset because of something that happened unexpectedly?” and “In the last month, how often have you felt that things were going your way?” This adaptation is characterized by good psychometric properties: (a) internal consistency which has been tested in a group of 120 adults obtaining a Cronbach’s alpha of 0.86; it indicates an adequate internal consistency for all questions with the overall scale score; (b) reliability over time (test/retest), and has been determined in a sample of 30 young adult university students within 2 days and 4 weeks intervals obtaining a Cronbach’s alpha of Rho = 0.90 and Rho = 0.72, respectively [38]. Scores for the four positively stated items (Items 4, 5, 7, 8) were reversed. The responses to the 10 items were then summed to create a psychological stress score. Individual scores on the PSS-10 can range from 0 to 40; the higher the scale score, the more likely the individual will perceive a higher level of psychological stress [37]. Scores ranging from 0–13 would be considered low stress; 14–26 moderate stress, and 27–40 high perceived stress [39].

The family, peer, and school environments were assessed on the basis of participants’ responses to the questions: (i) family: “How pressured do you feel by the high expectations from parents for your academic performance?”, (ii) peers: “How pressured do you feel by your class/team mates or other people your age try to get you to do something which you do not want to do?”, (iii) school-related stress: a generic stress “How pressured do you feel by the schoolwork you have to do?” And specific school stress “How pressured do you feel by upcoming tests; by participating in class; by a lack of support from the teacher?” The response options available were not at all (1), a little (2), somewhat (3), and a lot (4). The responses were recoded from 0 to 3, a higher score indicating more perceived pressure. The scores were divided into tertials: low, moderate, and high perceived levels of pressure.

Study participants were also asked to select on the test anxiety list any of the psychosomatic symptoms they usually experience before this stressful event.

### 2.4. Data Analysis

The prime objective of interest was to determine the set of factors that affect the likelihood of experiencing a high level of perceived stress (outcome) by adolescent students. For that, logistic regression analysis (LRA) was applied as the main statistical method. The dependent variable in the LRA was a dichotomous stress level: high (1) vs. low-to-moderate stress (0). Factors known or suspected to be the exposure of stress were chosen a priori for the analysis. They included: socio-demographic characteristics (gender, chronological age at the time of examination, type of school, and place of residence), height category and weight status as indicators of adolescent health and quality of diet, and environmental stressors related to three social environments: school (generic school-related stress and its specific causes: upcoming tests anxiety, participating in class, and a lack of support from teachers), the peer and parental relationships. Initially, an unadjusted association between high levels of perceived stress and each of the covariate variables was evaluated in bivariate relationships. Gender was found to be a significant predictor of high perceived stress; therefore, in the next step, gender-stratified adjusted analyses were performed using multiple logistic regression analysis (MLRA). The MLRA initially included all variables with a significance of *p*-value less than 0.05 in the univariate analysis. The odds ratio was used as a measure of the association between two and among multiple variables. A final explanatory model with a subset and relative odds (OR) of the factors associated with high level of stress was obtained using a stepwise procedure with backward elimination and rejection criterion of the *p*-value greater than 0.05.

Descriptive information on gender differences was based mainly on categorical variables and Pearson’s chi-square test. Student’s *t*-test was used for continuous variables.

To evaluate the importance (power) of statistically significant differences, their substantive significance (effect size) was calculated using Cohen’s guidelines and rules of thumb to qualify the magnitude of an effect size [40]. The Cohen’s *d* effect size index expressed in terms of Hedges’ *g* for groups with different sample size was used as a measure of effect size for difference between the means of two groups (boys and girls) and expressed in standard deviation units. The following suggested benchmark values for categorization into small 0.20 ≤ *d* < 0.50, medium 0.50 ≤ *d* < 0.80, and large *d* ≥ 0.8 effects were used. For odds ratio, the benchmarks defined were small 1.5 ≤ OR < 2.5, medium 2.5 ≤ OR < 4.3 and large OR ≥ 4.3 effects [40,41].

Statistical analyses were run using the STATISTICA version 13.3 data analysis software systems (StatSoft Inc., Tulsa, OK, USA). All significance tests comprised two-way determinations. A value of *p* < 0.05 was considered statistically significant.

## 3. Results

### 3.1. Major Characteristics of the Sample

Complete data were obtained from an ethnically homogeneous group (European Whites) of 1036 female and 810 male adolescents, mean age 15.75; SD = 1.76 years in boys and 15.93; SD = 1.69 years in girls. The total response rate was 97.4% and 95.6% for girls and boys, respectively. Table 1 gives sample distribution of the candidate predictor factors of perceived stress stratified by gender.

Place of residence, type of school, and body height category were almost evenly split among boys and girls. Large, statistically significant gender differences were found in the weight status and environmental stressors. Overall, a far greater proportion of girls than boys were underweight, and this difference was seen across three grades of thinness. Almost every second girl and every third boy experienced school-induced stress and its specific causes such as upcoming test and participation in class. A higher proportion of boys than girls were overweight and obese and felt pressures of peer relationships and high expectations from parents.

### 3.2. Perceived Stress Scores on the PSS-10

The overall PSS-10 score averaged 14.76 (SD = 6.85, 95% CI: 14.25–15.27) for boys compared to 19.19 (SD = 6.95, 95% CI: 18.75–19.64) for girls. The effect size of this difference was moderate (Hedges’ *g* = 0.641, 95% CI: 0.547–0.736). Furthermore, girls had higher scores in perceived stress across all 10 items of the PSS-10 than boys (Table 2). This result was statistically significant (most *p*-values less than 0.0001). However, effect sizes of these differences were mostly small. Hedges’ *g* values varied from 0.222 (95% CI: 0.126–0.319) for the Felt confident item to 0.414 (95% CI 0.316–0.511) for the Being upset item. The estimated strength of sex differences was moderate (medium size effect) for three other items, Nervous and stressed (Hedges’ *g* = 0.592; 95% CI: 0.494–0.691), Could not cope (Hedges’ *g* = 0.557; 95% CI: 0.459–0.655), and couldn’t overcome (Hedges’ *g* = 0.511; 95% CI: 0.413–0.609).

### 3.3. Prevalence of High Perceived Stress Levels

Prevalence of the most reported high perceived level, shown in Table 3, was associated with moderate levels of peer (25.5%) and parental (23.8%) pressures, high levels of stress from school (20.6%), and its specific causes—upcoming tests anxiety (20.8%) and participating in class (18.3%). There was also a substantial gender difference, with 17.8% of girls against 5.9% of boys reporting high perceived stress levels. It is worth noting that the prevalence of high stress was similar in both types of school and the difference was statistically not significant (20.7% and 24.9% for lower secondary school and upper secondary school, respectively). It means that school equally generated a stressful environment regardless of its type. Another finding showed that life in the countryside generated a high level of perceived stress amounting to 18.6% of our total sample. Interestingly, this proportion was greater than that of adolescents living in large cities. However, the difference between rural and three levels of urbanization was not statistically significant. The prevalence of high perceived stress was almost three-fold higher in older adolescents compared to their younger counterparts (15.4% vs. 5.8% in 18 and 13 years of age). The prevalence of high perceived stress was also significantly differentiated by health indicators, body height, and weight status. Adolescents shorter and taller than the average height peers experienced high perceived stress levels significantly more frequently (18.3%, and 10.9% vs. 8.6% for shorter and taller, respectively). Being underweight or overweight/obese compared to normal weight peers, predisposed adolescents to report high perceived stress level more frequently (17.2%, and 11 vs. 6.1%, for overweight/obese and underweight, respectively).

### 3.4. Candidate Predictor Factors Associated with High Level of Perceived Stress

Table 4 presents results of the univariate logistic regression analysis examining all of the possible combinations of the candidate predictor variables (stressors) associated with a high level of perceived stress. The analysis showed that all the stressors considered, except for the type of school and place of residence, were significantly associated with a high level of perceived stress. Direct associations were found for gender, with girls being over 3 times more likely than boys to experience perceived stress at high level (OR = 3.22; 95% CI: 2.15–5.82); age, with 13 years of age as reference category; weight status for overweight/obese adolescents and normal weight status as reference category; school-induced stress and its specific causes: upcoming tests, participating in class and lack of support from teachers; peer pressures and parental high expectations (low level of each stressor exposure as reference category). The only inverse association (odds ratio less than one) was observed between high perceived stress level and underweight (three grades of thinness) status (normal weight status as reference category) and adolescent height with taller than the average height group as reference category.

### 3.5. The Best Subset of Factors That Predict High Level of Perceived Stress across Gender

The best subset of high perceived stress predictors was determined from the dataset after adjusting for all candidate predictor factors but the type of school and place of residence and for each gender separately (Table 5). The finding showed that the best set of predictors shaping high level of perceived stress was slightly different in both genders.

In boys, the best subset of high perceived stress predictors included age, school-induced stress, peer and parental pressures, and lack of support from teachers. In girls, it included age, school-induced stress, stressful school events such as upcoming tests anxiety and participating in class, thinness and obesity, peer and parental pressures.

The odds of high perceived stress increased with each year of increasing age and by the age of 18 years, was almost three times in boys (OR = 2.82; 95% CI: 1.91–5.27) and over three times in girls (OR = 3.18; 95% CI: 1.29–7.81) greater than in 13-year-old counterparts.

Adolescents exposed to school-related stress were 4.5 times (boys) and 6.2 times (girls) more likely to experience a high level of perceived stress than those not exposed at all or only at low levels (OR = 4.45; 95% CI: 3.11–6.36 for boys and OR = 6.22; 95% CI: 4.18–7.59 for girls).

Adolescent boys exposed to high peer pressure were over four times and girls were over three times more likely to be stressed at a high level than their not exposed counterparts (OR = 4.15; 95% CI: 2.21–5.77 for boys and OR = 3.14; 95% CI: 2.35–4.97 for girls).

Similarly, those who were exposed to continuous high expectations from parents were over (boys), and almost (girls), three times more likely to experience high perceived stress levels as compared to their counterparts not exposed to parental stress (OR = 3.47; 95% CI: 2.06–4.72 for boys and OR = 2.63; 95% CI: 1.75–4.09 for girls).

While girls were stressed by abnormal (elevated or lowered) weight status, boys were stressed by their concern about height. The odds of high perceived stress level were almost three times greater in overweight/obese girls than in normal weight counterparts (OR = 2.63; 95% CI: 1.75–4.09). The likelihood of experiencing high perceived stress level increased 1.25 times with decreased value of BMI (thinness grade 3) giving OR = 0.80; 95% CI: 0.60–1.04. Boys shorter than the average height in group were considerably (almost twice) more likely than their taller counterparts to report high perceived stress levels (OR = 1.82; 95% CI: 1.09–2.51).

Furthermore, specific school-related stressful events were significant predictors of high perceive stress level only in girls. Those exposed to exam/test anxiety at a high level were almost two times more likely than their not exposed or at low level peers to experience high perceived stress (OR = 1.82; 95% CI: 1.20–3.05). A similar result was found for stress generated by participating in the classroom activities (OR = 1.84; 95% CI: 1.53–2.34).

While girls were more affected by school-related stressful events, boys experienced a high perceived stress level when exposed to a lack of support of the teacher (OR = 1.74; 95% CI: 1.01–2.94).

**Figure 1:**
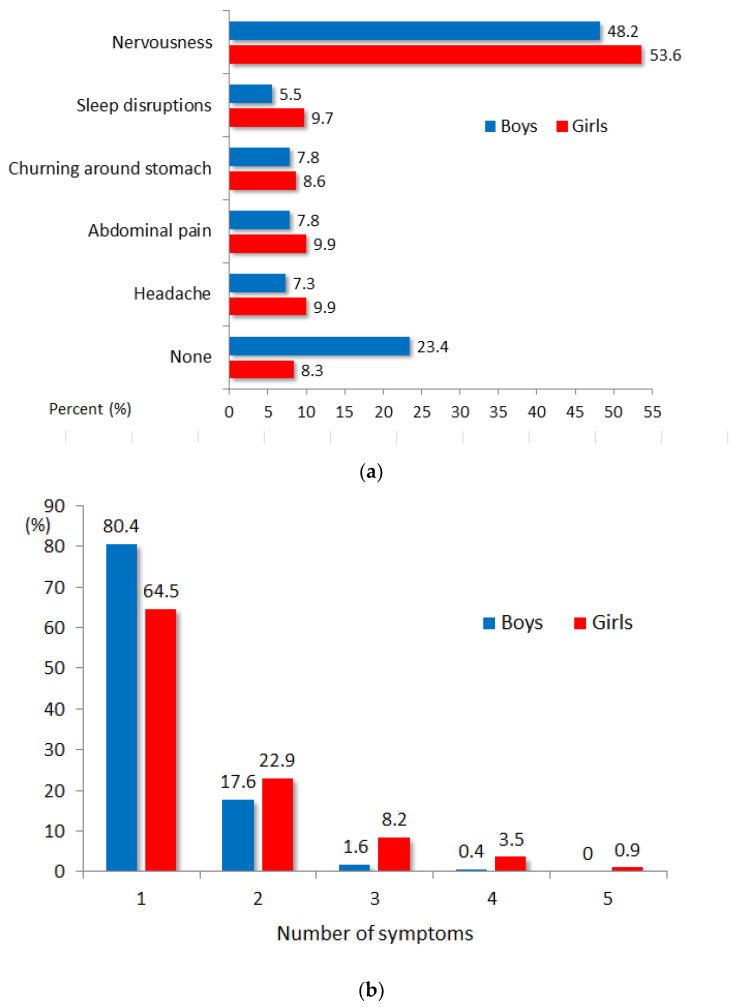
Frequency of psychosomatic symptoms evoked by exam/tests stress in adolescent students: (**a**) Specific symptoms; (**b**) Number of symptoms.

The patterning of statistically significant gender differences in perceived stress included also psychosomatic symptoms associated with exam/test anxious worry (Figure 1). Overall, 91.7% of girls compared to 76.6% of boys reported any of the symptoms. Nervousness was the most common symptom reported by both girls (53.6%) and boys (48.2%) followed by headache, abdominal pain, sleep disruptions, and churning around the stomach (Figure 1a). Gender difference was statistically significant (Chi-square = 36.49, df = 5, *p* < 0.001). Relative to boys, girls were much more likely to experience multiple symptoms of exam/tests anxiety (Figure 1b) and this gender difference was statistically significant too (Chi-square = 58.30, df = 4, *p* < 0.001). For example, three symptoms were about 4 times (8.2% in girls versus 1.6% in boys) and four symptoms almost 9 times (3.5% in girls versus 0.4% in boys) more frequently reported by girls than boys.

## 4. Discussion

Utilizing a transactional model of stress and coping and a large sample of adolescents aged 13 to 18 years, the present study provides the first, as we believe, data that show perceived stress in relation to school environment, peers, and parents among Polish youths. Results of this study may contribute in several ways to the public health research literature on stress in adolescence and the pediatric clinical practice.

One of the key findings revealed that of all the examined factors, school-induced stress was the most powerful predictor (with large effect size) of a high perceived stress level. This was true for both genders regardless of their rural/urban settings and the type of school they attended. Several studies showed similar results [15,16,42,43,44].

School is recognized as a central developmental context for the academic and socio-emotional development of adolescent students. School can be seen as adolescents’ main workplace, an environment where they face a wide range of ongoing normative stressors such as academic demands (tests, exams, class participation) strengthened by high parental expectations [15,42,43]. School is also an important institution for the socialization of adolescents in modern societies shaped by teacher–student interpersonal relationships, relational work and likeability among the same-aged peers [44]. Exposure to anxiety or stress induced from school affects adolescent well-being. School well-being plays a significant role in current and future well-being, psychosomatic problems, and academic achievements [15].

The concept of stage-environment fit, a theoretical outgrowth of the ecological system perspective, suggests that a poor fit between individual changes during adolescence (e.g., pubertal changes in physique, desires for autonomy and a greater role in decision-making; a heightened need for positive relationships with adults outside the family, and sensitivity to peer evaluation) and contextual levels (e.g., greater teacher control and discipline; less personal and positive teacher–student relationships; fewer opportunities for student decision-making and choice, and more emphasis on ability assessment and social comparison) may hinder his/her ability to cope with and adapt to a stressful school environment [45]. If this fit is unsuitable, the individual may experience maladaptation. If it is adequate (optimal), the individual’s motivation, behavior, and overall mental and physical health may be facilitated. Our findings indicated a large overall effect of school environment on perceived stress. This is interpreted as of practical significance, thus suggesting that school-based intervention programs targeting adolescent students might mitigate stress and improve their mental health status and well-being [46].

Gender has proven to be a medium effect factor of perceived stress with girls being more likely than boys to experience systematically higher perceived stress levels, score higher on a perceived stress scale, and report more frequently psychosomatic symptoms of exam anxiety.

The exact gender-related differences in prevalence rate and general score vary among adolescents across several different cultures and social contexts [47,48,49,50,51]. In line with our finding, a study by Murberg and Bru [52] found gender difference in school stress vulnerability, with girls being likely to experience more stress related to school achievement (in the present study, not only generic stress from school but also participating in class and upcoming test anxiety) while boys experienced more stress from relational conflicts with teachers, parents, and peers at school. Despite the heterogeneous results across studies, the well-established gender difference in perceived stress is believed to emerge at adolescence and often passes through to later periods of life [53].

The reasons behind the gender gap are less well understood. The explanations suggested include potential gender differences in sociocultural milieu [54] and biological sex differences in physiological [55] and neurobiological [56] mechanisms underlying every part of the stress process. There is also much evidence on gender determined differences in emotional reactions to stress, in particular, that related to establishing and maintaining interpersonal relationships in the adolescent period, with girls showing higher levels of stress than boys [57].

Bale and Epperson [58] in their systematic literature review have demonstrated that apart for the prenatal organizational window to sex differences in long-term programing, there are limited differences in early postnatal life until the onset of puberty. This critical moment in life is associated with gender-related changes in psychological stress. In line with the focal theory of change [59], the magnitude of perceived stress depends on the accumulation of experience from previous stages of life. Both the accumulation and new exposures to stressful environmental stimuli may result in much higher stress in adolescence than any other period of life. Furthermore, the current study showed that prevalence of high perceived stress level and overall magnitude of stress increased with each increasing year in both boys and girls. This finding is broadly consistent with other studies [50,53]. Seiffge-Krenke and colleagues, however, found that most adolescents experienced high levels of stress during early adolescence up to the age of 15, after which it began decreasing [60].

Studies evaluating the association between the BMI weight status and perceived stress have yielded conflicting results. Our analysis revealed a significant positive association between being overweight/obese and a high level of perceived stress and negative association between being thin and perceived stress, but only in girls. This finding corroborates that of the HELENA-CSS study [61] but is inconsistent with reports indicating overweight/obese boys and girls being directly associated with perceived stress [62,63], and null results found in our previous study conducted among Polish adolescents where perceived stress was measured in terms of depressive symptoms [64]. The results of the EAT Study have suggested that impairment in the emotional well-being of overweight/obese adolescents is mediated by weight-related body dissatisfaction in both boys and girls and during early and late adolescence [21]. In contrast to these findings, the 4-year longitudinal study of van Jaarsveld and colleagues [65] revealed that perceived stress in any year was not related prospectively to increases in waist or BMI, nor was there any evidence that higher stress over the whole period was associated with greater gains in waist or BMI.

Furthermore, inverse associations of body height with perceived stress (the shorter height, the greater stress), found in the present study only in boys, confirmed gender-oriented aspects of overall own body perception (body image) or body dissatisfaction. A growing body of literature in this area, including our previous studies, clearly suggests that girls almost exclusively report wanting to be thinner [66,67,68,69] whereas the majority of boys desire to be taller and more muscular [70,71]. Although less frequently addressed in the literature, height dissatisfaction is suggested to be associated with a higher drive for muscularity and seems to be a male-specific phenomenon [72].

### Study Limitations

This study has several limitations. First, the data were based on self-reports from adolescents and should be evaluated with reference to potential self-reporting bias. However, self-reporting of perceived stress is commonly used in psychological studies largely because it provides a more appropriate measure of actual levels of stress experienced by individuals than external counting of potential stressors [73]. Second, the cross-sectional design did not allow us to make conclusions regarding causality but was methodologically appropriate for solving the research question, to assess the prevalence of high perceived stress level and to determine the best subset of its predictors. Despite these limitations, the strengths of this study were the conceptual framework that underpinned our study (the transactional model of stress and coping), use of validated psychometric instruments, population-based cohort data of non-clinical adolescent students, the relatively large sample size, high response rate, a multivariate approach to integrate multiple factors hypothesized to be associated with high perceived stress level and measuring the strength of these relationships and differences. With the advantages of systematic sampling, the results of this study can be generalized to the entire cohort of Polish adolescents.

## 5. Conclusions

Results of the present study provide valuable insights into the impact of stressful school setting, peer and parental pressures on perceived stress of Polish adolescents. As the significant effects of school-induced stress and gender differences on perceived stress were large and medium, the challenge to researcher and practitioners is clear. Gender differences in psychological reaction to diverse stressors, both individual and environment, should be the subject of further detailed investigation. The sources of stress reported to be most common by adolescents should be discussed with parents, school principals and health policy makers to develop and implement school-based stress reduction programs for adolescents.

## Figures and Tables

**Table 1:** Candidate predictor factors of perceived stress: sample distribution according to gender.

Variable	Boys *n* = 810 (43.8%)	Girls *n* = 1036 (56.2%)	*p*-Value ^1^
Age, Mean (SD) (years)	15.75 (1.76)	15.93 (1.69)	0.083
Place of residence, *n* (%)			0.062
Rural areas	173 (21.4)	268 (25.9)	
Small town	213 (26.3)	205 (19.8)	
Mid-sized city	251 (30.9)	298 (28.8)	
Large city	173 (21.4)	265 (25.5)	
Type of school, *n* (%)			0.276
Lower secondary school	415 (51.2)	507 (48.9)	
Upper secondary school	395 (48.8)	529 (51.1)	
Body height category (percentile), *n* (%)			0.685
Shorter, <the 25th	125 (15.4)	191 (18.4)	
Average, ≥the 25th and ≤the 75th	387 (47.8)	492 (47.5)	
Taller, >the 75th	298 (36.8)	353 (34.1)	
Weight status (BMI kg/m^2^), *n* (%)			0.004
Thinness grade 3	1 (0.1)	3 (0.3)	
Thinness grade 2	6 (0.8)	14 (1.4)	
Thinness grade 1	53 (6.5)	105 (10.1)	
Normal/healthy weight	602 (74.3)	782 (75.5)	
Overweight	129 (15.9)	110 (10.6)	
Obesity class 1	19 (2.4)	22 (2.1)	
School-induced stress, *n* (%)	306 (37.8)	463 (44.7)	<0.001
Upcoming test anxiety, *n* (%)	276 (34.1)	440 (42.5)	0.015
Participating in class, *n* (%)	301 (37.2)	495 (47.8)	0.003
Lack of support from teachers, *n* (%)	179 (22.1)	189 (18.3)	0.048
Peer pressure, *n* (%)	125 (15.4)	106 (10.2)	0.025
Parental pressure, *n* (%)	242 (29.9)	205 (19.8)	0.029

^1^ Gender differences: *p*-values represent results of independent samples Student’s *t*-test for continuous variables and Pearson’s chi-square test for categorical variables.

**Table 2:** Gender differences in mean scores across all ten items of the Perceived Stress Scale, PSS-10.

PSS-10 Item	Mean (SD)		*t*	*p*-Value	Hedges’ *g*
	Boys	Girls			(95% CI)
Item 1 Being upset	2.01 (0.98)	2.42 (1.00)	5.82	<0.0001	0.41 (0.32–0.51)
Item 2 Unable to control	1.47 (1.13)	1.90 (1.20)	5.13	<0.0001	0.37 (0.27–0.46)
Item 3 Nervous and stressed	2.09 (1.04)	2.73 (1.11)	8.59	<0.0001	0.59 (0.49–0.69)
Item 4 Felt confident *	1.19 (1.05)	1.41 (0.94)	3.09	0.002	0.22 (0.13–0.32)
Item 5 Going your way *	1.26 (0.95)	1.63 (0.89)	5.74	<0.0001	0.40 (0.31–0.50)
Item 6 Could not cope	1.45 (1.06)	2.05 (1.09)	7.75	<0.0001	0.56 (0.46–0.65)
Item 7 Control irritations *	1.22 (1.03)	1.48 (0.96)	3.72	0.0002	0.26 (0.17–0.36)
Item 8 On top of things *	1.52 (1.01)	1.90 (0.95)	5.62	<0.0001	0.39 (0.29–0.49)
Item 9 Been angered	1.80 (1.21)	2.24 (1.04)	5.64	<0.0001	0.39 (0.29–0.49)
Item 10 Couldn’t overcome	1.38 (1.11)	1.95 (1.12)	7.02	<0.0001	0.51 (0.41–0.61)

Note: PSS-10, 10-item Perceived Stress Scale; * Reverse coded item.

**Table 3:** Prevalence of high perceived stress level experienced by adolescent students across levels of candidate predictor factors.

Predictor Factor	High Stress %	*p*-Value	Predictor Factor	High Stress %	*p*-Value
Socio-demographic stressors			Environmental stressors		
Gender		<0.001	Type of school		0.219
Boys	5.9		Lower Secondary School	20.7	
Girls	17.8		Upper Secondary School	24.9	
Age (years) ^a^		<0.001	School-induced stress		<0.001
13	5.8		Low	2.8	
14	9.5		Moderate	14.8	
18	15.4		High	20.6	
Place of residence		0.065	Upcoming tests anxiety		0.017
Rural areas	18.6		Low	9.5	
Small town	9.7		Moderate	20.8	
Mid-sized city	6.9		High	16.1	
Large city	14.2		Participating in class		0.028
Interpersonal stressors			Low	9.2	
Peer pressure		<0.001	Moderate	14.0	
Low	10.2		High	18.3	
Moderate	25.5		Teachers’ lack of support		0.024
High	19.8		Low	1.5	
Parental pressure		<0.001	Moderate	7.6	
Low	10.2		High	12.9	
Moderate	23.8		Health indicators		
High	17.3		Weight status (BMI kg/m^2^)		0.046
			Normal weight	6.1	
			Underweight	11.4	
			Overweight/Obesity	17.2	
			Height category (percentile)		0.048
			<the 25th	18.3	
			≥the 25th and ≤the 75th	8.6	
			>the 75th	10.9	

Note: *p*-values represent results of Pearson’s chi-square tests for categorical variables. ^a^ Age range varies from 13 to 18 years.

**Table 4:** Bivariate associations between high level of perceived stress and candidate predictor factors in adolescent study participants: results of logistic regression analysis, unadjusted odds ratio and 95% confidence interval.

Predictor Factor	OR (95% CI)	Predictor Factor	OR (95% CI)
Gender		School-induced stress	
Boys (Ref.)	1	Low (Ref.)	1
Girls	3.22 (2.15; 5.82)	Moderate	3.84 (2.27; 4.49)
*p*-*trend* < 0.001		High	4.01 (3.45; 6.02)
Age (years) ^a^		*p*-*trend* < 0.001	
13 (Ref.)	1	Upcoming tests anxiety	
14	1.29 (1.13; 1.47)	Low (Ref.)	1
18	4.57 (2.08; 7.04)	Moderate	1.81 (1.19; 2.77)
*p*-*trend* < 0.001		High	2.25 (1.76; 3.08)
Place of residence		*p*-*trend* = 0.005	
Rural areas (Ref.)	1	Participating in class	
Small town	0.93 (0.84; 1.07)	Low (Ref.)	1
Mid-sized city	0.89 (0.79; 1.01)	Moderate	1.61 (1.20; 2.34)
Large city	0.85 (0.58; 1.23)	High	1.92 (1.61; 2.50)
*p*-*trend* = 0.325		*p*-*trend* = 0.038	
Type of school		Teachers’ lack of support	
Lower Secondary (Ref.)	1	Low (Ref.)	1
Upper Secondary	1.46 (0.95; 2.24)	Moderate	1.66 (0.71; 2.91)
*p*-*trend* = 0.084		High	1.92 (1.03; 3.42)
Peer pressure		*p*-*trend* = 0.027	
Low (Ref.)	1	Weight status (BMI kg/m^2^)	
Moderate	2.55 (1.14; 3.71)	Normal weight (Ref.)	1
High	3.01 (1.81; 4.99)	Underweight	0.83 (0.70; 1.02)
*p*-*trend* < 0.001		Overweight/Obesity	1.56 (1.42; 1.73)
Parental pressure		*p*-*trend* = 0.032	
Low (Ref.)	1	Height category (percentile)	
Moderate	1.59 (1.04; 2.44)	>the 75th (Ref.)	1
High	2.85 (1.92; 3.93)	≥the 25th and ≤the75th	1.29 (0.93; 1.59)
*p*-*trend* < 0.001		<the 25th	1.69 (0.99; 2.44)
		*p*-*trend* = 0.048	

Note: ^a^ Age range varies from 13 to 18 years.

**Table 5:** The best subset of predictor factors affecting the likelihood of experiencing high level of perceived stress in adolescent boys and girls: results of multivariate logistic regression analysis, adjusted odds ratio, and 95% confidence interval.

Boys ^a^		Girls ^a^	
Predictor Factor	OR (95% CI)	Predictor Factor	OR (95% CI)
Age (years) ^b^		Age (years)	
13 (Ref.)	1	13 (Ref.)	1
14	1.38 (0.98; 1.92)	14	1.21 (1.04; 1.40)
18	2.82 (1.91; 5.27)	18	3.18 (1.19; 7.81)
*p*-*trend* = 0.045		*p*-*trend* = 0.011	
School-induced stress		School-induced stress	
Low (Ref.)	1	Low (Ref.)	1
Moderate	3.11 (2.62; 4.89)	Moderate	3.58 (2.45; 6.08)
High	4.45 (3.11; 6.36)	High	6.22 (4.18; 7.59)
*p*-*trend* = 0.002		*p*-*trend* < 0.001	
Teachers’ lack of support		Upcoming tests anxiety	
Low (Ref.)	1	Low (Ref.)	1
Moderate	1.46 (0.63; 2.42)	Moderate	1.41 (1.09; 2.35)
High	1.74 (1.01; 2.94)	High	1.82 (1.20; 3.05)
*p*-*trend* = 0.035		*p*-*trend* = 0.020	
Peer pressure		Participating in class	
Low (Ref.)	1	Low (Ref.)	1
Moderate	3.32 (2.11; 4.52)	Moderate	1.56 (1.14; 2.35)
High	4.15 (2.21; 5.77)	High	1.84 (1.53; 2.34)
*p*-*trend* < 0.001		*p*-*trend* = 0.043	
Parental pressure		Peer pressure	
Low (Ref.)	1	Low (Ref.)	1
Moderate	2.32 (1.78; 3.82)	Moderate	2.82 (1.98; 4.57)
High	3.47 (2.06; 4.72)	High	3.14 (2.35; 4.97)
*p*-*trend* < 0.009		*p*-*trend* = 0.019	
Height category (percentile)		Parental pressure	
>the 75th (Ref.)	1	Low (Ref.)	1
≥the 25th and ≤the75th	1.35 (0.98; 1.87)	Moderate	1.99 (1.58; 3.05)
<the 25th	1.82 (1.09; 2.51)	High	2.63 (1.75; 4.09)
*p*-*trend* = 0.047		*p*-*trend* = 0.022	
		Weight status (BMI kg/m^2^)	
		Normal weight (Ref.)	1
		Underweight	0.80 (0.60; 1.04)
		Overweight/Obesity	2.63 (1.75; 4.09)
		*p*-*trend* = 0.020	

Note: ^a^ Adjusted odds ratio after controlling for all candidate predictor variables. Final most parsimonious subset of factors affecting the likelihood of high perceived stress was obtained using a stepwise backward elimination method and rejection criterion of the *p*-value greater than 0.05. ^b^ Age range varies from 13 to 18 years.

## Data Availability

This study does not report any data.

## References

[1] Bogin B., Smith B.H. (1996). Evolution of the human life cycle. Am. J. Hum. Biol..

[2] Del Giudice M., Angeleri R., Manera V. (2009). The juvenile transition: A developmental switch point in human life history. Dev. Rev..

[3] Cameron N., Bogin B. (2012). Human Growth and Development.

[4] Gomes F.V., Rincón-Cortés M., Grace A.A. (2016). Adolescence as a period of vulnerability and intervention in schizophrenia: Insights from the MAM model. Neurosci. Biobehav. R.

[5] Graber J.A. (2013). Pubertal timing and the development of psychopathology in adolescence and beyond. Horm. Behav..

[6] Anniko M.K., Boersma K., Tillfors M. (2019). Sources of stress and worry in the development of stress-related mental health problems: A longitudinal investigation from early- to mid-adolescence. Anxiety Stress. Copin..

[7] McEwen B.S. (2007). Physiology and neurobiology of stress and adaptation: Central role of the brain. Physiol. Rev..

[8] Wulsin A.C., Wick-Carlson D., Packard B.A., Morano R., Herman J.P. (2016). Adolescent chronic stress causes hypothalamo-pituitary-adrenocortical hypo-responsiveness and depression-like behavior in adult female rats. Psychoneuroendocrino.

[9] Lindholdt L., Labriola M., Andersen J.H., Kjeldsen M.-M.Z., Obel C., Lund T. (2021). Perceived stress among adolescents as a marker for future mental disorders: A prospective cohort study. Scand. J. Public Health.

[10] Schneiderman N., Ironson G., Scott D.S. (2005). Stress and health: Psychological, behavioral, and biological determinants. Annu. Rev. Clin. Psychol..

[11] Lazarus R.S., Folkman S. (1984). Stress, Coping and Adaptation.

[12] Phillips A.C., Gellman M.D., Turner J.R. (2018). Perceived stress. Encyclopedia of Behavioural Medicine.

[13] Piekarska J. (2020). Determinants of perceived stress in adolescence: The role of personality traits, emotional abilities, trait emotional intelligence, self-efficacy, and self-esteem. Adv. Cogn. Psychol..

[14] Liu X., Zhao Y., Li J., Dai J., Wang X., Wang S. (2020). Factor structure of the 10-item perceived stress scale and measurement invariance across genders among chinese adolescents. Front. Psychol..

[15] Nygren K., Hagquist C. (2019). Self-reported school demands and psychosomatic problems among adolescents-changes in the association between 1988 and 2011?. Scand. J. Public Health.

[16] Pascoe M.C., Hetrick S.E., Parker A.G. (2020). The impact of stress on students in secondary school and higher education. Int. J. Adolesc. Youth.

[17] OECD (2017). Schoolwork-Related Anxiety, in PISA 2015 Results (Volume III): Students’ Well-Being.

[18] Jaureguizar J., Bernaras E., Bully P., Garaigordobil M. (2018). Perceived parenting and adolescents’ adjustment. Psicol. Refl. Crít..

[19] Topor D.R., Keane S.P., Shelton T.L., Calkins S.D. (2010). Parent involvement and student academic performance: A multiple mediational analysis. J. Prev. Interv. Community.

[20] Little B., Zeigler-Hill V., Shackelford T.K. (2020). The Role of peers in personality development. Encyclopedia of Personality and Individual Differences.

[21] Mond J., van den Berg P., Boutelle K., Hannan P., Neumark-Sztainer D. (2011). Obesity, body dissatisfaction, and emotional well-being in early and late adolescence: Findings from the project EAT Study. J. Adolesc. Health.

[22] Van Vuuren C.L., Wachter G.G., Veenstra R., Rijnhart J.J.M., van der Wal M.F., Chinapaw M.J.M., Busch V. (2019). Associations between overweight and mental health problems among adolescents, and the mediating role of victimization. BMC Public Health.

[23] Mills J.S., Amy Shannon A., Hogue J. (2017). Beauty, Body Image, and the Media.

[24] Zametkin A.J., Zoon C.K., Klein H.W., Munson Z. (2004). Psychiatric aspects of child and adolescent obesity: A review of the past 10 years. J. Am. Acad. Child. Adolesc. Psychiatry.

[25] Patalay P., Gage S.H. (2019). Changes in millennial adolescent mental health and health-related behaviours over 10 years: A population cohort comparison study. Int. J. Epidemiol..

[26] World Health Organization Fact Sheet—Adolescent Mental Health in the WHO European Region 2018. https://www.euro.who.int/data/assets/pdf_file/0005/383891/adolescent-mh-fs-eng.pdf.

[27] Liu R.T., Alloy L.B. (2010). Stress generation in depression: A systematic review of the empirical literature and recommendations for future study. Clin. Psychol. Rev..

[28] Inchley J., Currie D., Budisavljevic S., Torsheim T., Jåstad A., Cosma A., Kelly C., Arnasson A.M. (2020). Spotlight on Adolescent Health and Well-Being. Findings from the 2017/2018 Health Behaviour in School-Aged Children (HBSC) Survey in Europe and Canada. International Report. Volume 2. Key Data.

[29] UNICEF Innocenti (2020). Worlds of Influence: Understanding What Shapes Child Well-Being in Rich Countries.

[30] World Medical Association Declaration of Helsinki (2001). Ethical principles for medical research involving human subjects. Bull. World Health Organ. Int. J. Public Health.

[31] Lemeshow S., Hosmer D.W., Klar J., Lwanga S.K. (1990). Adequacy of Sample Size in Health Studies.

[32] Tillé Y. (2006). Sampling Algorithms.

[33] Knussmann R. (1988). Anthropologie, Handbuch der Vergleichenden Biologie des Menschen.

[34] Kułaga Z., Litwin M., Tkaczyk M., Palczewska I., Zajączkowska M., Zwolińska D., Krynicki T., Wasilewska A., Moczulska A., Morawiec-Knysak A. (2011). Polish 2010 growth references for school-aged children and adolescents. Eur. J. Pediatr..

[35] Cole T.J., Bellizzi M.C., Flegal K.M., Dietz W.H. (2000). Establishing a standard definition for child overweight and obesity worldwide: International survey. Br. Med. J..

[36] Cole T.J., Flegal K.M., Nicholls D., Jackson A.A. (2007). Body mass index cut offs to define thinness in children and adolescents: International survey. Br. Med. J..

[37] Cohen S., Kamarck T., Mermelstein R. (1983). A global measure of perceived stress. J. Health Soc. Behav..

[38] Juczyński Z., Ogińska –Bulik N. (2009). Narzędzia Pomiaru Stresu i Radzenia Sobie ze Stresem.

[39] Cohen S., Williamson G.M., Spacapan S., Oskamp S. (1998). Perceived stress in a probability sample of the United States. The Social Psychology of Health: Claremont Symposium on Applied Social.

[40] Cohen J. (1988). Statistical Power Analysis for the Behavioral Sciences.

[41] Lenhard W., Lenhard A. Calculation of Effect Sizes. Psychometrica: Dettelbach, Germany. https://www.psychometrica.de/effect_size.html.

[42] Wuthrich V.M., Jagiello T., Azzi V. (2020). Academic stress in the final years of school: A systematic literature review. Child. Psychiatry Hum. Dev..

[43] Çelik E. (2019). Stress regarding academic expectations, career exploration, and school attachment: The mediating role of adolescent–parent career congruence. Aust. J. Career Dev..

[44] Kiuru N., Wang M.T., Salmela-Aro K., Kannas L., Ahonen T., Hirvonen R. (2020). Associations between adolescents’ interpersonal relationships, school well-being, and academic achievement during educational transitions. J. Youth Adolesc..

[45] Eccles J.S., Roeser R.W. (2011). Schools as developmental contexts during adolescence. J. Res. Adolesc..

[46] Van Loon A.W.G., Creemers H.E., Beumer W.Y., Okorn A., Vogelaar S., Saab N., Miers A.C., Westenberg P.M., Asscher J.J. (2020). Can schools reduce adolescent psychological stress? A multilevel meta-analysis of the effectiveness of school-based intervention programs. J. Youth Adolesc..

[47] Gupta A., Sharma R.P., Goyal P., Midha T. (2010). Perceived stress among adolescents—A cross-sectional study in high school students of Kanpur city. Indian J. Mat. Child. Health.

[48] Kim K., Park H. (2016). Gender differences in the association between self-reported stress and cigarette smoking in Korean adolescents. Tob. Induc. Dis..

[49] Pinto A.A., Claumann G.S., Medeiros P., Barbosa R.M.D.S.P., Nahas M.V., Pelegrini A. (2017). Association between perceived stress in adolescence, body weight and romantic relationships. Rev. Paul. Pediatr..

[50] Thaker R., Verma A. (2014). A study of perceived stress and coping styles among mid adolescents. Natl. J. Physiol. Pharm. Pharmacol..

[51] Srivastava P., Kumar P., Kiran M. (2015). Perceived stress and self-esteem among school going adolescents: A gender perspective. J. Disabil. Manag. Rehabil..

[52] Murberg T.A., Bru E. (2004). School-related stress and psychosomatic symptoms among Norwegian adolescents. School Psychol. Int..

[53] Aldwin C., Folkman S. (2011). Stress and coping across the lifespan. Oxford Library of Psychology. The Oxford Handbook of Stress, Health, and Coping.

[54] Andersen J., Lindholdt L., Winding T., Lund T., Labriola M. (2017). Perceived stress among adolescents is socially determined. Eur. J. Public Health.

[55] Condon E.M. (2018). Chronic Stress in Children and Adolescents: A Review of Biomarkers for Use in Pediatric Research. Biol. Res. Nurs..

[56] Blakemore S.-J. (2012). Imaging brain development: The adolescent brain. NeuroImage.

[57] Rudolph K.D. (2002). Gender differences in emotional responses to interpersonal stress during adolescence. J. Adolesc. Health.

[58] Bale T.L., Epperson C.N. (2015). Sex differences and stress across the lifespan. Nat. Neurosci..

[59] Coleman J.C., Hurrelmann K., Engel U. (1989). The focal theory of adolescence: A psychological perspective. The Social World of Adolescents: International Percepctives. Prevention and Intervention in Childhood and Adolescence.

[60] Seiffge-Krenke I., Aunola K., Nurmi J.-E. (2009). Changes in stress perception and coping during adolescence: The role of situational 604 and personal factors. Child Dev..

[61] De Vriendt T., Clays E., Maes L., De Bourdeaudhuij I., Vicente-Rodriguez G., Moreno L.A., Nagy E., Molnár D., Ortega F.B., Dietrich S. (2012). European adolescents’ level of perceived stress and its relationship with body adiposity—The HELENA Study. Eur. J. Public Health.

[62] Hamaideh S.H., Al-Khateeb R.Y., Al-Rawashdeh A.B. (2010). Overweight and obesity and their correlates among Jordanian adolescents. J. Nurs. Scholarsh..

[63] Askari J., Hassanbeigi A., Khosravi H.M., Malek M., Hassanbeigi D., Pourmovahed Z., Alagheband M. (2013). The relationship between obesity and depression. Proc. Soc. Behav. Sci..

[64] Trambacz-Oleszak S., Krzyżaniak A., Szafrańska-Komarowska I., Kaczmarek M. (2018). Body weight status is not a predictive factors of depressive symptoms in Polish adolescents aged 13–18 years. Anthropol. Rev..

[65] van Jaarsveld C.H.M., Fidler J.A., Steptoe A., Boniface D., Wardle J. (2009). Perceived stress and weight gain in adolescence: A longitudinal analysis. Obesity.

[66] Kaczmarek M., Trambacz-Oleszak S. (2016). The association between menstrual cycle characteristics and perceived body image: A cross-sectional survey of Polish female adolescents. J. Biosoc. Sci..

[67] Xie B., Unger J.B., Gallaher P., Johnson C.A., Wu Q., Chou C. (2010). Overweight, body image, and depression in Asian and Hispanic adolescents. Am. J. Health Behav..

[68] Wilkosz M., Chen J., Kenndey C., Rankin S. (2011). Body dissatisfaction in California adolescents. J. Am. Acad. Nurse Pract..

[69] Xanthopoulos M., Borradaile K., Hayes S., Sherman S., Vander Veur S., Grundy K., Foster G. (2011). The impact of weight, sex, and race/ethnicity on body dissatisfaction among urban children. Body Image.

[70] Tylka T.L. (2011). Refinement of the tripartite influence model for men: Dual body image pathways to body change behaviors. Body Image.

[71] Dakanalis A., Timko A., Madeddu F., Volpato C., Clerici M., Riva G., Zanetti A.M. (2015). Are the male body dissatisfaction and drive for muscularity scales reliable and valid instruments?. J. Health Psychol..

[72] Baker J.H., Higgins Neyland M.K., Thornton L.M., Runfola C.D., Larsson H., Lichtenstein P., Bulik C. (2019). Body dissatisfaction in adolescent boys. Dev. Psychol..

[73] Nielsen N.R., Zhang Z.F., Kristensen T.S., Netterstrøm B., Schnohr P., Grønbaek M. (2005). Selfreported stress and risk of breast cancer: Prospective cohort study. BMJ.

